# Monitoring of Insecticide Resistance in *Anopheles culicifacies* in Twelve Districts of Madhya Pradesh, Central India (2017–2019)

**DOI:** 10.1155/2022/4404027

**Published:** 2022-01-05

**Authors:** Ashok K. Mishra, Praveen K Bharti, Gyan Chand, Aparup Das, Himanshu Jayswar, Manju Rahi, Kamaraju Raghavendra

**Affiliations:** ^1^ICMR-National Institute of Research in Tribal Health, Jabalpur, India; ^2^State Malaria Program Officer, Directorate of Health Services Bhopal, Bhopal, India; ^3^Indian Council of Medical Research (ICMR), New Delhi, India; ^4^National Institute of Malaria Research, New Delhi, India

## Abstract

**Background:**

Indoor residual spraying (IRS) and long-lasting insecticidal nets (LLINs) are malaria vector control measures used in India, but the development of insecticide resistance poses major impediments for effective vector control strategies. As per the guidelines of the National Vector Borne Disease Control Programme (NVBDCP), the study was conducted in 12 districts of Madhya Pradesh to generate data on insecticide resistance in malaria vectors.

**Methods:**

The susceptibility tests were conducted on adult *An. culicifacies* as per the WHO standard technique with wild-caught mosquitoes. The blood-fed female mosquitoes were exposed in 3 to 4 replicates on each occasion to the impregnated papers with specified discriminating dosages of the insecticides (DDT: 4%, malathion: 5%, deltamethrin: 0.05%, and alphacypermethrin: 0.05%), for one hour, and mortality was recorded after 24-hour holding.

**Results:**

*An. culicifacies* was found resistant to DDT 4% in all the 12 districts and malathion in 11 districts. The resistance to alphacypermethrin was also observed in two districts, and possible resistance was found to alphacypermethrin in seven districts and to deltamethrin in eight districts, while the vector was found susceptible to both deltamethrin and alphacypermethrin in only 3 districts.

**Conclusion:**

*An. culicifacies* is resistant to DDT and malathion and has emerging resistance to pyrethroids, alphacypermethrin, and deltamethrin. Therefore, regular monitoring of insecticide susceptibility in malaria vectors is needed for implementing effective vector management strategies. However, studies to verify the impact of IRS with good coverage on the transmission of disease are required before deciding on the change of insecticide in conjunction with epidemiological data.

## 1. Introduction

Malaria is a major public health problem in India, contributing to about 89% of incidence from South East Asia [1]. Five Indian states are responsible for transmission of more than 70% of malaria in the country of which Madhya Pradesh is the fifth highly malarious state which contributes about 5% of total malaria cases [2]. *Anopheles culicifacies* is the main malaria vector in rural and periurban areas in India contributing to about 65% of annual malaria transmission [3]. Insecticide-based vector control interventions currently in use in India include indoor residual spraying (IRS) and long-lasting insecticidal nets (LLINs) [4]. One of the major impediments for effective vector control is the development of resistance in vectors to the insecticides which are used in public health sprays. Presently, three insecticides, DDT (organochlorine), malathion (organophosphate), and mostly synthetic pyrethroids, are used in IRS and LLINs. *An. culicifacies* has shown resistance to DDT [5–7] and malathion [8, 9] and also reduced susceptibility to synthetic pyrethroids in a few areas including in Madhya Pradesh and Chhattisgarh [10–14]. This study was undertaken as a task force project under the aegis of the Indian Council of Medical Research. Based on epidemiological data and geographic ecosystems, the National Vector Borne Disease Control Programme (NVBDCP) selected 12 districts of Madhya Pradesh to generate data on insecticide resistance in malaria vectors (Table 1).

There are 50 districts in Madhya Pradesh with a population of about 60 million, including 12 million tribal populations. The state consists of sparsely settled forested hills with a 31% forested area and serves as a reservoir for intense perennial malaria transmission [15]. In the present investigation, we monitored the insecticide susceptibility status of *An. culicifacies* in 12 districts of Madhya Pradesh against commonly used insecticides in the public health system.

## 2. Materials and Methods

### 2.1. Study Area

The susceptibility tests against *An. culicifacies* were carried out in 12 districts of Madhya Pradesh located in the northern, eastern, western and southern parts of the state, namely, districts Umaria, Singrauli, Anuppur, Panna, Tikamgarh, Hoshangabad, Khargone, Dhar, Alirajpur, Bhind, Datia, and Shivpuri, from July 2017 to July 2019. In the districts, three to seven villages in two to three CHCs (about 1% of total villages in the district) in different terrains, i.e., hilltop, plain, foothill, and forest terrains were selected for the studies (Table 2). Anuppur, Umaria, and Bhind were under DDT indoor spray and Singrauli, Panna, Hoshangabad, Khargone, Dhar, Alirajpur, and Shivpuri were under alphacypermethrin (synthetic pyrethroid) indoor spray. In districts Tikamgarh and Datia, there was no routine indoor spray for the last >20 years due to low malaria prevalence (<2 API). However, in the year 2016, 26 villages in the district of Tikamgarh received focal sprays of DDT. In five districts, viz., Panna, Anuppur, Singrauli, Alirajpur, and Dhar, long-lasting insecticide-treated nets were distributed (Table 2). However, all the districts were proposed for LLIN distribution by the year 2019.

### 2.2. Insecticide Susceptibility Tests

Susceptibility tests were conducted on adult *An. culicifacies* following essentially the WHO standard procedures using the kit and method [16]. Wild-caught mosquitoes were collected from different resting sites (indoors-human dwellings/cattle sheds and outdoors) and preferably blood-fed female mosquitoes [17] and identified based on morphological characters [18] in the selected villages of the districts in the different months from 2017 to 2019 (Table 2). The collected mosquitoes were brought to the laboratory for testing in cloth cages wrapped with wet towels. Female mosquitoes were exposed in 3 to 4 replicates on each occasion to the WHO impregnated papers with specified discriminating dosages of the insecticides (DDT: 4%, malathion: 5%, deltamethrin: 0.05%, and alphacypermethrin: 0.05%), with respective insecticide controls for comparison (two replicates) for one hour, and mortality was recorded after 24-hour holding. The tests were repeated within 2 or 3 days in different villages of different terrains in each district. Cartons with wet towels at the bottom were used to conduct the tests to maintain the ambient temperature of 26 ± 2°C and the RH of 70–80% [19]. Mortality after 24 hrs of holding period was recorded [20]. Percent mortality was calculated separately for the test and control replicates using the following formula: % observed mortality = number of dead mosquitoes × 100/number of mosquitoes tested.

If the mortality in control replicates is between 5% and 20%, the test mortality was corrected with the control mortality using Abbott's formula [21]. In cases where the mortality in the controls exceeded 20%, the test was discarded: % corrected mortality = (% test mortality – % control mortality) × 100/(100 – % control mortality).

According to the WHO criteria [20], mosquito species that show on exposure to the diagnostic dosage of a given insecticide a mortality rate of 98 to 100% are designated as “susceptible,” <90% as “confirmed resistance,” and between 90% and 98% as “possible resistance.”

## 3. Results

**Figure 1:**
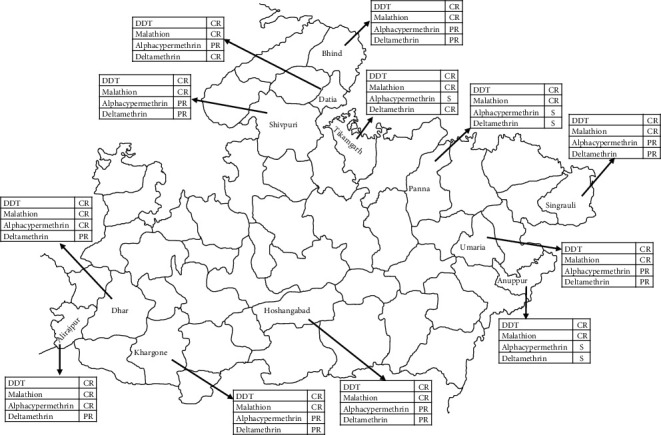
Map of Madhya Pradesh showing study districts and insecticide susceptibility status of *An. culicifacies.*

Results of the susceptibility tests carried out in 12 districts are given in Table 3. *An. culicifacies* was found resistant to DDT in all the districts with a % mortality rate ranging from 7.6 to 60% and resistant to malathion in 11 districts (62 to 87%) (Figure 1).

In district Datia, species showed possible resistance to malathion, registering 93% mortality. Resistance to alphacypermethrin was observed in Dhar and Alirajpur districts where % mortality was 84.2 and 87.6, respectively, and tests were repeated after 6 months, and the mortality was 82.9 and 85.7% indicating no variation in mortality (Table 3).

*An. culicifacies* was reported susceptible to pyrethroids, viz., alphacypermethrin and deltamethrin in 3 districts, i.e., Anuppur, Panna, and Tikamgarh (98.1 to 100.0% mortality), while in Datia it was susceptible to deltamethrin (100% mortality).

*An. culicifacies* was possibly resistant to alphacypermethrin in 7 districts, viz., Singrauli, Umaria, Hoshangabad, Khargone, Datia, Shivpuri, and Bhind, with mortality ranging from 90.5 to 97.0%. However, to deltamethrin, possible resistance in *An*. *culicifacies* was observed in 8 districts, viz., Singrauli, Umaria, Hoshangabad, Khargone, Alirajpur, Dhar, Shivpuri, and Bhind where mortality registered was between 93.3 and 97% (Table 3).

The terrain-wise pooled data of 12 districts (Table 4) showed similar susceptibility status in all 4 terrains, i.e., plain, foothill, hilltop, and forest areas except for deltamethrin. The species was possibly resistant with registered mortality of 96.7%, 94.3%, and 96.4%, respectively, in plain, foothill, and hilltop terrains, whereas in forest terrain the species was susceptible to deltamethrin with 98% mortality. However, the difference in observed mortalities was within a range of 2–4% indicating the population to be near susceptible or possibly resistant.

*An. culicifacies* showed resistance to DDT and malathion in all the terrains with the observed % mortality rate to DDT in the range of 14.5 to 23.4 and to malathion in the range of 67.0 to 74.4%. Possible resistance to alphacypermethrin was observed in all 4 terrains with a mortality rate in the range of 91.3 to 95.4%.

Based on the spray history in last 10 years in different districts, districts were categorized into three groups: group A-IRS with pyrethroids, 7 districts, viz., Panna, Hoshangabad, Singrauli, Khargone, Dhar, Alirajpur, and Shivpuri; group B-IRS with DDT, 3 districts, viz., Anuppur, Umaria, and Bhind; and group C-without IRS, 2 districts, viz., Tikamgarh and Datia (Table 5). *An. culicifacies* was found resistant to DDT and malathion registering low % mortality rates for DDT of 16.2, 15.6, and 37.6% in groups A, B, and C, respectively, while increased % mortality rates were registered for malathion at 69.2, 68.3, and 88.8%, respectively. To pyrethroid alphacypermethrin, the species showed possible resistance in groups A and B with % mortality in the range of 90.2 and 96.2, respectively, but was susceptible in group C with 98.5% mortality. Statistical analysis of mortalities against alphacypermethrin between the sprayed group (A) and the no spray group (C) was highly significant (chi sq. = 15.36, *p* < 0.0001) and with the DDT sprayed group (B) (chi. Sq. = 11.15, *p*, 0.001) and no significance was seen in alphacypermethrin mortality when compared with the no spray (C) and the DDT sprayed (B) group (chi. Sq. = 2.44, *p*=0.118). The species showed possible resistance to deltamethrin with % mortality in the range of 95.2 and 97.1% in groups A and B, respectively, but was completely susceptible in group C.

## 4. Discussion

Insecticide resistance is becoming a limiting factor for effective malaria vector control for national programmes worldwide, especially in view of the committed elimination of malaria in this decade by 2030. Presently, about 125 species of mosquitoes are documented to show resistance to one or more insecticides.

Raghavendra et al. [22] reviewed the status of insecticide resistance among the major malaria vectors in India in the last quarter century (1991–2016) based on the available information from published and unpublished reports. Resistance to DDT in *An. culicifacies* is widespread in the country [5, 6], and resistance to malathion is widespread in the districts in the states of Maharashtra [8], Gujrat [23, 24], Andhra Pradesh [24], Uttar Pradesh [9], and Madhya Pradesh [13]. There are a few reports of resistance to synthetic pyrethroids in various parts of the country [10–14]. Resistance to malathion was detected in five districts of Andhra Pradesh, nine districts of Odisha, and possible resistance in two districts of Jharkhand, 4 districts of Odisha, and 4 districts of West Bengal. *An. culicifacies* was found susceptible to malathion in two districts of Jharkhand and six districts of Odisha, resistant to deltamethrin in four districts of Andhra Pradesh, with possible resistance in 10 districts of Odisha, and susceptible to deltamethrin in some districts of Odisha, Jharkhand, and West Bengal [25].

In the present study, in Madhya Pradesh, *An. culicifacies* the main malaria vector was found resistant to DDT 4% in all the 12 districts surveyed and resistant to malathion in 11 districts, except in Datia district where the species is reported possibly resistant. The species was reported resistant to alphacypermethrin in two districts Dhar and Alirajpur. This vector was found susceptible to both deltamethrin and alphacypermethrin in three districts, i.e., Anuppur, Panna, and Tikamgarh. Possible resistance was found to alphacypermethrin in seven districts, namely, Singrauli, Umaria, Hoshangabad, Datia, Shivpuri, Bhind, and Khargone, and to deltamethrin in eight districts, viz., Singrauli, Umaria, Hoshangabad, Khargone, Alirajpur, Dhar, Shivpuri, and Bhind. Thus, the species was resistant to DDT and malathion in all the districts while it was mostly possible resistant to pyrethroids.

It may be stated that DDT has been sprayed in these areas in surveyed districts since the inception of the national malaria control activities in the early 1950s. Decreased mortality in *An. culicifacies* to pyrethroids was found in areas that received alphacypermethrin IRS in the last 5–10 years. In all the areas, the species in different districts have shown resistance to DDT and malathion. However, in areas without pyrethroid indoor spray, the species registered possible resistance and were susceptible to pyrethroids, alphacypermethrin, and deltamethrin in the range of 96.2 to 100%. Malathion was not sprayed regularly in these areas and the observed resistance to malathion could be due to possible selection by its use in agriculture/forestry in the absence of its use in public health sprays but needs further investigation.

To date, DDT, malathion, deltamethrin, alphacypermethrin, and lambda cyhalothrin are the most commonly used insecticides for vector control in public health in India, and other pyrethroid insecticides, namely, cyfluthrin and bifenthrin, are also recommended for use in antimalaria sprays [26]. Deltamethrin and alphacypermethrin impregnated LLINs are in extensive use in India in different states of the country, and Madhya Pradesh is receiving the LLINs in 2019 in all endemic districts.

The resistance in mosquitoes may develop due to changes in their enzyme systems resulting in more rapid detoxification or sequestration of the insecticide or due to mutations in the target site preventing the insecticide target site interaction [27]. Spraying of insecticides without proper understanding of the prevailing resistance mechanism may lead to increased vector resistance and failure of vector control intervention. In India, end-point replacement of insecticides is practiced after failure of control of a given class of insecticide resulting in multiple resistance in malaria vectors [28].

## 5. Conclusion

Results of the present study in 12 districts of Madhya Pradesh indicate that *An. culicifacies* is reported resistant in all the districts to DDT and to malathion, while to pyrethroids, alphacypermethrin, and deltamethrin the species is reported mostly possible resistant. Owing to the dynamics of development of resistance as evidenced from the above study, there is a need for regular monitoring of insecticide susceptibility in malaria vectors for implementing effective disease vector management strategies. However, studies to verify the impact of IRS with good coverage on transmission of disease are needed before deciding on a change of insecticide in conjunction with epidemiological data [26]. In addition, insecticide molecules with novel modes of action belonging to new classes of insecticides and insecticide mixtures such as neonicotinoid and pyrrole including carbamate class of insecticides for IRS and LLIN interventions are in development/trials. These molecules need to be adapted for vector control in our country to keep the date for elimination. Furthermore, it needs to be emphasized that the regulatory norms being followed for the introduction of interventions need to be reviewed for faster introduction. This will facilitate to preserve the gains achieved so far and pave the way for a faster impact on the transmission of malaria and disease control, ultimately leading to malaria elimination by date.

## Figures and Tables

**Table 1:** Epidemiological situation in the districts selected for the insecticide monitoring study.

Districts	Year	Population	BSE	+VE	PF	ABER	API	SPR	PF%
Dhar	2015	2364759	348960	4328	1949	15.00	2.00	1.00	45.00
	2016	2412054	320115	2100	765	13.00	1.00	1.00	36.00
Hoshangabad	2015	1343272	197175	1672	665	14.68	1.24	0.85	39.0
	2016	1372137	197608	1139	445	14.42	0.83	0.58	39.0
Anuppur	2015	811306	79769	2007	1280	10.00	3.00	3.00	64.00
	2016	776248	79504	1226	764	10.00	2.00	2.00	62.00
Panna	2015	1078217	167850	1332	242	16.00	1.00	1.00	18.00
	2016	1099781	13969	1582	479	13.00	1.00	1.00	30.00
Tikamgarh	2015	1534632	152233	1336	18	9	0.87	0.88	1.35
	2016	1564028	185300	1327	38	11.8	0.85	0.72	2.86
Shivpuri	2016	1884582	220925	3885	576	12.00	2.00	2.00	15.00
	2017	1922690	201781	1648	93	11.00	1.00	1.00	6.00
Datia	2016	868232	90424	456	8	44.00	2.00	2.00	7.00
	2017	885586	113899	506	3	10.00	0.50	0.50	2.00
Alirajpur	2016	800942	122482	1602	713	15	2	1	44
	2017	810614	84331	704	302	10	1	1	43
Bhind	2016	1880870	218174	3424	51	11.60	1.82	1.57	1.49
	2017	1918488	191213	1925	11	9.97	1.00	1.01	0.57

*Note.* The epidemiological data of three districts (Singrauli, Khargone, and Umaria) were not available at the time of study. BSE: blood smear examination, +VE: number of malaria positive cases, PF: *Plasmodium falciparum*, ABER: annual blood examination rate, API: annual parasite incidence, SPR: slide positivity rate, and PF%: *Plasmodium falciparum* percentage.

**Table 2:** Profile of study areas including the vector control measures and the period of study.

S No.	Districts	Location	Insecticide used for IRS	LLIN distributed (yes or no)	No. of study villages	Ecotype of villages	Period of surveys
1	Anuppur	East	DDT	Yes	6	Plain, foothill, hilltop	Sept 2017
2	Panna	North east	SP	Yes	6	Plain, foothill, hilltop	Oct 2017
3	Tikamgarh	North	No spray	No	3	Plain, forest	Oct 2017
4	Singrauli	East	SP	Yes	6	Plain, foothill, hilltop	Oct 2017
5	Umaria	East	DDT	No	6	Plain, foothill, hilltop	July 2017
6	Hoshangabad	South	SP	Yes	6	Plain, foothill, hilltop	Apr 2018
7	Khargone	South	SP	No	6	Plain, foothill, forest	Sept 2018
8	Alirajpur	West	SP	Yes	6	Plain, foothill, forest	Dec 2018 and Jul 2019
9	Dhar	West	SP	Yes	6	Plain, foothill, forest	Dec 2018 and Jul 2019
10	Shivpuri	North	SP	No	7	Plain, foothill, hilltop	Feb 2019
11	Datia	North	No spray	No	6	Plain, forest	Feb 2019
12	Bhind	North	DDT	No	7	Plain, foothill	Jul 2019

SP = synthetic pyrethroids: alphacypermethrin

**Table 3:** Susceptibility status of *An. culicifacies* to discriminatory dosages of DDT, malathion, alphacypermethrin, and deltamethrin in 12 districts of Madhya Pradesh.

Insecticide-%	Districts	No. of mosquitoes exposed		Dead 24 hr		Mortality (%)^*∗*^	Susceptibility status^*∗∗*^
		Exp	Control	Exp	Control		
DDT-4%	Anuppur	105	60	17	1	16.2	CR
	Panna	105	45	13	1	12.4	CR
	Singrauli	105	45	19	0	18.1	CR
	Tikamgarh	105	30	17	0	16.2	CR
	Umaria	105	45	15	0	14.3	CR
	Hoshangabad	105	60	8	2	7.6	CR
	Khargone	120	60	15	1	12.5	CR
	Alirajpur	105	45	8	1	7.6	CR
	Dhar	120	45	21	0	17.5	CR
	Datia	100	45	60	1	60	CR
	Shivpuri	100	55	41	0	41	CR
	Bhind	105	45	17	0	16.2	CR

Malathion-5%	Anuppur	105	60	68	1	64.8	CR
	Panna	105	45	77	0	73.3	CR
	Singrauli	105	45	74	2	70.5	CR
	Tikamgarh	105	30	89	0	84.8	CR
	Umaria	105	45	80	0	76.2	CR
	Hoshangabad	105	60	76	2	72.4	CR
	Khargone	120	60	78	0	65	CR
	Alirajpur	105	45	61	0	58.1	CR
	Dhar	120	45	78	0	65	CR
	Datia	100	45	93	2	93	PR
	Shivpuri	100	55	87	2	87	CR
	Bhind	105	45	67	0	63.8	CR

Alphacypermethrin-0.05%	Anuppur	105	60	103	2	98.1	S
	Panna	105	45	104	0	99	S
	Singrauli	105	45	95	1	90.5	PR
	Tikamgarh	105	30	105	0	100	S
	Umaria	105	45	100	1	95.2	PR
	Hoshangabad	105	60	101	0	96.2	PR
	Khargone	120	60	112	1	93.3	PR
	Alirajpur	105	45	92	2	87.6	CR
	Dhar	120	45	101	1	84.2	CR
	Datia	100	45	97	2	97	PR
	Shivpuri	100	55	93	2	93	PR
	Bhind	105	45	100	0	95.2	PR

Deltamethrin-0.05%	Anuppur	105	60	103	2	98.1	S
	Panna	105	45	104	0	99	S
	Singrauli	105	45	98	1	93.3	PR
	Tikamgarh	105	30	105	0	100	S
	Umaria	105	45	101	1	96.2	PR
	Hoshangabad	105	60	100	0	95.2	PR
	Khargone	120	60	116	1	96.7	PR
	Alirajpur	105	45	101	2	96.2	PR
	Dhar	120	45	112	1	93.3	PR
	Datiya	100	45	100	2	100	S
	Shivpuri	100	55	97	2	97	PR
	Bhind	105	45	102	0	97.1	PR

^*∗*^The control mortality in all districts in all insecticides was either <5.0. ^*∗∗*^ CR = confirmed resistant, PR = possible resistant, and S = susceptible.

**Table 4:** Terrain-wise grouped insecticide susceptibility data in *An. culicifacies*.

Type of terrain	Insecticide	No. of mosquitoes exposed		Mortality in 24 hr		Mortality (%)	Susceptibility status^*∗*^
		Exp	Control	Exp	Control		
Plain	DDT	600	285	116	3	19.3	CR
	Malathion	600	285	442	4	73.7	CR
	Alphacypermethrin	600	285	548	10	91.3	PR
	Deltamethrin	600	285	580	10	96.7	PR
Forest	DDT	295	120	69	0	23.4	CR
	Malathion	295	120	213	1	72.2	CR
	Alphacypermethrin	295	120	274	0	92.9	PR
	Deltamethrin	295	120	289	0	98.0	S
Foothill	DDT	400	190	58	2	14.5	CR
	Malathion	400	190	268	1	67.0	PR
	Alphacypermethrin	400	190	366	4	91.5	PR
	Deltamethrin	400	190	377	4	94.3	PR
Hilltop	DDT	195	75	40	2	20.5	CR
	Malathion	195	75	145	4	74.4	CR
	Alphacypermethrin	195	75	186	0	95.4	PR
	Deltamethrin	195	75	188	0	96.4	PR

^*∗*^CR = confirmed resistance, PR = possible resistance, and S = susceptible.

**Table 5:** Susceptibility status of *An. culicifacies* in the districts grouped under different categories based on IRS.

Villages with different insecticide sprays	Insecticide	No. of mosquitoes exposed		No. of mosquitoes dead		% mortality	Susceptibility status^*∗*^
		Test	Control	Test	Control		
Group A—pyrethroid IRS since last 5–10 years and earlier with DDT IRS-7 districts (Singrauli, Panna, Hoshangabad, Khargone, Dhar, Shivpuri, and Alirajpur)	DDT	970	445	157	5	16.2	CR
	Malathion	970	445	671	7	69.2	CR
	Alphacypermethrin	970	445	875	9	90.2	PR
	Deltamethrin	970	445	923	9	95.2	PR

Group B—DDT spray since last 5–10 years—3 districts (Anuppur, Bhind, and Umaria)	DDT	315	105	49	1	15.6	CR
	Malathion	315	105	215	1	68.3	CR
	Alphacypermethrin	315	105	303	3	96.2	PR
	Deltamethrin	315	105	306	3	97.1	PR

Group C—no spray since last >20 years—2 districts (Tikamgarh and Datia)	DDT	205	75	77	1	37.6	CR
	Malathion	205	75	182	2	88.8	CR
	Alphacypermethrin	205	75	202	2	98.5	S
	Deltamethrin	205	75	205	2	100.0	S

^*∗*^CR = confirmed resistant, PR = possible resistant, and S = susceptible.

## Data Availability

All the data are reported in the manuscript. The hardcopy of the data is available from the corresponding author on reasonable request.
